# Dilute Polymerization of Aniline on PDMS Substrate via Surface Modification Using (3-Aminopropyl)Triethoxysilane for Stretchable Strain Sensor

**DOI:** 10.3390/s22072741

**Published:** 2022-04-02

**Authors:** Chang-Joo Yim, Ji-Yeon Choy, Hae-Kyung Youi, Jung-Hoon Hwang, Eun-Bee Jo, Jun-Ho Lee, Hyun-Seok Kim

**Affiliations:** Division of Electronics and Electrical Engineering, Dongguk University-Seoul, Seoul 04620, Korea; akreal@dongguk.edu (C.-J.Y.); jiyeon9711@dongguk.edu (J.-Y.C.); yeahwooong@dgu.ac.kr (H.-K.Y.); dk2323@dgu.ac.kr (J.-H.H.); 2021120265@dgu.ac.kr (E.-B.J.); steve1211@dgu.ac.kr (J.-H.L.)

**Keywords:** strain sensor, conducting polymer, self-assembled monolayer, dilute polymerization, gauge factor

## Abstract

Stretchable strain sensors are capable of acquiring data when in contact with human skin or equipment and are widely used in wearable applications. Most strain sensors have tensile properties of less than 20% and have limitations regarding body motion linkage, complex sensor structure, and motion nonreliability. To address these problems, we developed a high tension and high sensitivity sensor with a gauge factor over 40 and tensile stress about 50%. Polydimethylsiloxane (PDMS) was selected as the flexible substrate to ensure tensile strength, and polyaniline (PANI) was used to measure the resistance changes in the sensor. In particular, problems regarding poor uniformity of PANI on PDMS were resolved by surface treatment of the PDMS, wherein PANI polymerization was performed sequentially after forming a self-assembled monolayer (SAM) on the PDMS substrate. O_2_ plasma and (3-aminopropyl)triethoxysilane were used to form the SAM. It is expected that this sensor can obtain stable characteristics even under high tensile stress through the evenly formed PANI films on the surface-treated PDMS substrate and may be used in various flexible sensor applications.

## 1. Introduction

Conductive polymers have the electrical and optical properties of metals as well as mechanical properties of original polymers; hence, extensive research is currently underway on their potential applications [1,2,3,4,5]. Polyaniline (PANI) has good electrical properties and high mechanical strength, thereby rendering it suitable for application to flexible-substrate-based wearable sensors [6,7,8]. Existing flexible substrate-based wearable sensors have been studied for various applications, such as electronic skin, flexible displays, health monitors, and energy harvesters; however, they are limited by several restrictions regarding use in practical applications owing to their lack of tensile properties [9,10,11]. Therefore, using PANI to develop stretchable sensors with excellent tensile properties and conductivity, such as strain-sensor-based electronic skin, is expected to have highly valuable applications [12,13].

PANI films can be fabricated easily on substrates by various chemical, electrochemical, template, and interfacial synthesis methods [14,15,16,17]. The chemical polymerization method, which uses redox reactions, is widely utilized owing to simplicity of synthesis [18,19]. However, variables such as the type of oxidant, synthesis temperature and time, and surface treatment must be considered carefully to control PANI’s shape, length, electrical characteristics, and yield [20,21]. In particular, the surface functional groups on a substrate are crucial for achieving high adhesive force, uniform coverage, and good electrical properties of PANI during the chemical polymerization process [22,23,24]. The present study is aimed at manufacturing a crack-based strain sensor by synthesizing a PANI film on a polydimethylsiloxane (PDMS) substrate via surface treatment through self-assembled monolayer (SAM) formation [22,24,25].

The gauge factor (GF) is an essential indicator for evaluating the performance of a strain sensor [26]. GF is defined as

(1) $$\mathrm{GF}=\frac{R/R_{0}}{L/L_{0}}$$

GF was expressed as the change in relative resistance with relative length (Equation (1)). *R*_0_ and *L*_0_ denote the resistance and the length in the initial state. *R* and *L* represent the resistance and length in the strain state. Generally, for metal thin films, the GF is as low as 2–5. Low GF indicates low sensor sensitivity, which means that a material with high GF is required for precise sensing.

Previously, several researchers have attempted PANI polymerization on various substrates with insufficient tensile properties, such as Si, PEN and PET [27,28]. In addition, a few studies have attempted PANI polymerization on tensile substrates such as PDMS [8,29]. It is not difficult to synthesize PANI films on PDMS substrates, but uniform and stable characteristics in manufacturing sensors are the greater concerns. Therefore, a simple process for synthesizing PANI films on self-assembled monolayer (SAM)-treated PDMS substrate is suggested in this study to ensure high GF and tensile properties. The suggested synthesis process is suitable for manufacturing inexpensive, flexible, sensitive, and reversible sensors.

## 2. Materials and Methods

### 2.1. Materials

PDMS used as the flexible substrate was made using a Si elastomer base and a curing agent (sylgard 184) purchased from Dow Corning Corporation (Midland, MI, USA). Aniline (≥99.5%) for PANI synthesis was purchased from Sigma-Aldrich (St. Louis, MO, USA), perchloric acid (70%) was purchased from Sigma-Aldrich (St. Louis, MO, USA), and ammonium persulfate (≥98%) was purchased from Sigma-Aldrich (St. Louis, MO, USA). Toluene (≥99.9%) for surface treatment and (3-aminopropyl)triethoxysilane (APTES, 99%) were both purchased from Sigma-Aldrich (St. Louis, MO, USA). Acetone and isopropanol (IPA) used for cleaning were purchased from J.T. Baker (Radnor, PA, USA).

### 2.2. Methods

The PDMS substrate synthesis process is as follows. PDMS is combined in a base to curing agent ratio of 10:1 in a 4-inch Petri dish and mixed thoroughly for 10 min; then, a treatment procedure involving vacuum for 5 min, release for 1 min, and waiting for 5 min was repeated three times in a vacuum desiccator to remove air bubbles. Thereafter, curing was performed at 100 °C for 48 min in an oven to complete the fabrication of bulk PDMS, followed by cooling and removal from the Petri dish [18]. Finally, the PDMS was cut using a mold made according to the ASTM D412 model [30,31,32].

The PDMS substrate was then subject to the following surface treatment process. First, the surface was cleaned with acetone, IPA, and deionized (DI) water for 1 min each and dried with nitrogen. Then, surface modifications are performed using O_2_ plasma under 100 W, 20 sccm, and 100 mTorr for 2 min. Thereafter, the PDMS surface treatment is completed by immersing the substrate in 10 mM APTES in toluene for 30 min. Finally, the substrate is naturally dried for 3 h.

**Figure 1:**
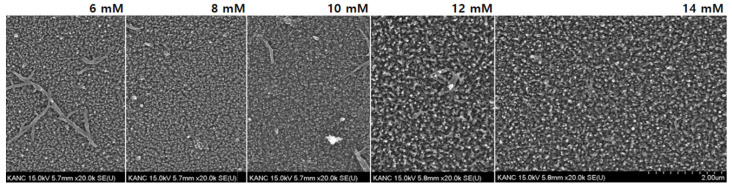
Scanning electron microscopy images of PANI with different mole concentrations of aniline.

PANI films are deposited on the PDMS surface by the dilute polymerization process. First, the concentration at which PANI can be most evenly formed on the surface was selected. The typical ratio of aniline to APS is 1.5 [18]. However, we tried to find the most suitable synthesis concentration of aniline in the range of 6–14 mM while fixing the APS concentration as shown in Figure 1. The density with 14 mM of aniline is sufficiently ensured and better as compared to other concentrations. Therefore, we prepare 14 mM of aniline solution and 9.33 mM APS solution in 1 M perchloric solution, followed by precooling for 30 min. The surface-treated PDMS substrate is attached to the beaker, and the prepared aniline solution is first added, followed by slow addition of the prepared APS solution. The beaker is then maintained at 0 °C for 24 h and stirred at 200 rpm. After completion of synthesis, the final product is cleaned in DI water and dried with a nitrogen gun. The various prepared samples are listed in Table 1. The sample 1 is a PANI on PDMS without any treatment. The sample 2 is a PANI on PDMS after O_2_ plasma treatment. The sample 3 is a PANI on PDMS after O_2_ plasma and APTES treatments. Circle and cross indicate whether or not they have gone through any treatment processes.

## 3. Results

### 3.1. SAM on PDMS

**Figure 2:**
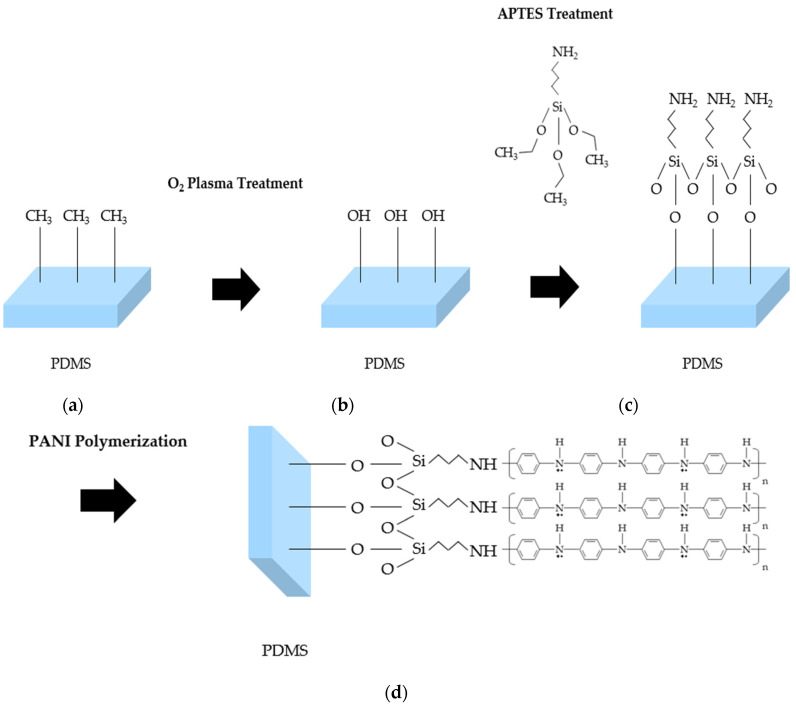
PDMS surface modification: (**a**) pristine PDMS; (**b**) O_2_ plasma treatment; (**c**) APTES treatment; (**d**) PANI polymerization after surface modification.

A SAM refers to an organic monolayer that is formed spontaneously on the surface of the substrate. The organic molecules of the SAM consist of three parts: the first part is the head group, which enables chemical adsorption of the molecules on the surface; the second part is the body group that enables ordered molecular film formation; the third part is the tail group that determines the chemical functional groups of the formed molecular film. Here, the amine group (NH_2_) is used as the tail group to ensure high deposition rate with PANI during the dilute polymerization process. Synthesis is performed using the same characteristics of the amine group (NH_2_) of PANI and the tail group of SAM. Figure 2 shows the process of synthesis of PANI after surface treatment. When PANI is synthesized on a substrate, aniline must be deposited in seed form for PANI growth. If this seed is nonuniformly deposited on the substrate, the final deposited product will also be nonuniform. Therefore, for PANI to grow uniformly on the substrate, it is noted that the surface of the substrate must also be chemically uniform. PDMS has a –CH_3_ functional group (Figure 2a) that is converted to an –OH group through O_2_ plasma treatment (Figure 2b). Thereafter, this functional group is replaced with the NH_2_ group to facilitate PANI synthesis through APTES treatment (Figure 2c). Finally, PANI polymerization is performed on the modified PDMS surface (Figure 2d). To grow a uniform PANI layer on the PDMS surface and increase the overall coverage, it is essential to form a SAM. Because the surface is not chemically uniform in the pristine PDMS, it is subjected to O_2_ plasma and then silane (here, APTES) treatments to form a chemically uniform surface capable of achieving good adhesion with PANI.

### 3.2. Scanning Electron Microscopy (SEM)

**Figure 3:**
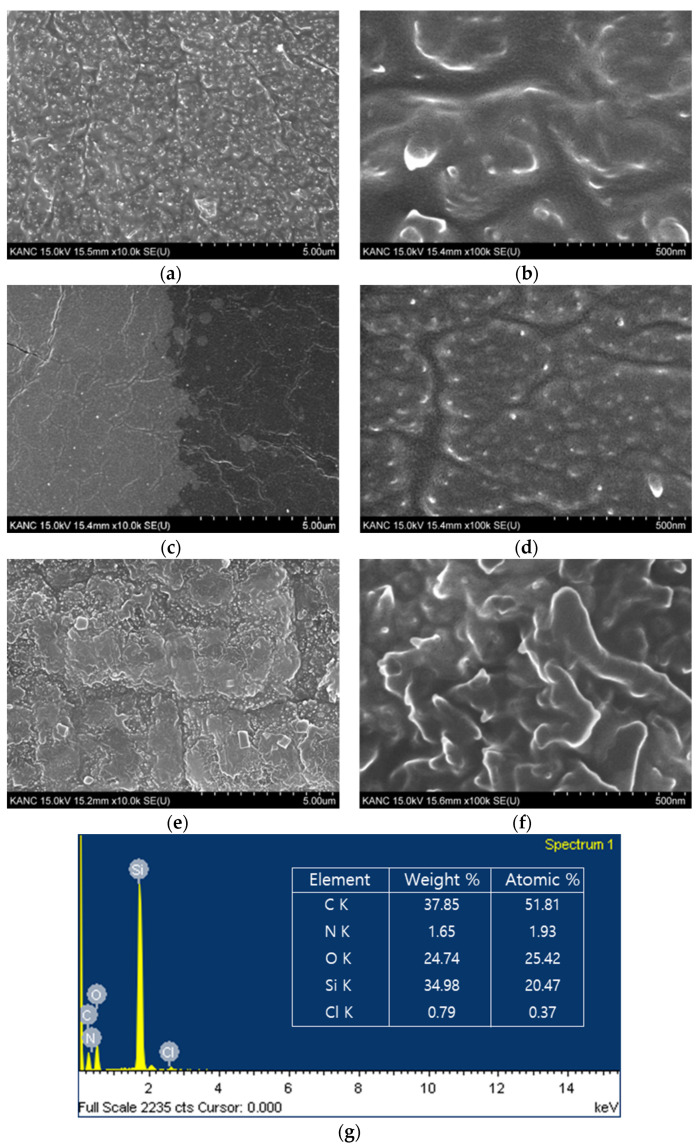
Scanning electron microscopy images of sample 1, 2 and 3 at 10 k and 100 k magnifications: (**a**,**b**) PANI on pristine PDMS; (**c**,**d**) PANI on PDMS after O_2_ plasma treatment; (**e**,**f**) PANI on PDMS after O_2_ plasma and APTES treatments; (**g**) energy-dispersive X-ray spectroscopy pattern and element composition of the PANI film (sample 3).

We selected PDMS as the substrate and confirmed PANI film growth based on surface treatment in the dilute polymerization process of PANI. The surface morphology and elemental composition were studied through SEM-coupled energy-dispersive X-ray spectroscopy (EDS) analysis. Figure 3a,b shows the growth of PANI on PDMS without any surface treatment (sample 1); it is seen that the PANI film was deposited on the PDMS surface such that the density was sufficiently secured. However, Figure 3c,d shows PANI growth after O_2_ plasma treatment of the PDMS (sample 2), and it is seen that the density is significantly less than that of PANI grown on pristine PDMS. This shows that synthesis is poor when PANI is grown immediately after O_2_ plasma treatment. The formation of –OH group on PDMS substrate alone proves that PANI is difficult to synthesize normally. Figure 3e,f shows that APTES treatment was performed immediately after O_2_ plasma treatment of the PDMS, followed by PANI growth (sample 3); here, the shape itself is not much different from the sample with PANI growth on pristine PDMS, but the density is sufficiently ensured, thickness is greater, and linkage is better. The EDS pattern of the sample 3 (Figure 3g) show several peaks for the PANI film, with carbon (C) and nitrogen (N) signals representing PANI, silicon (Si), and oxygen (O) signals derived from APTES surface treatment, and chloride (Cl) signals from perchloric acid.

### 3.3. Contact Angle

**Figure 4:**
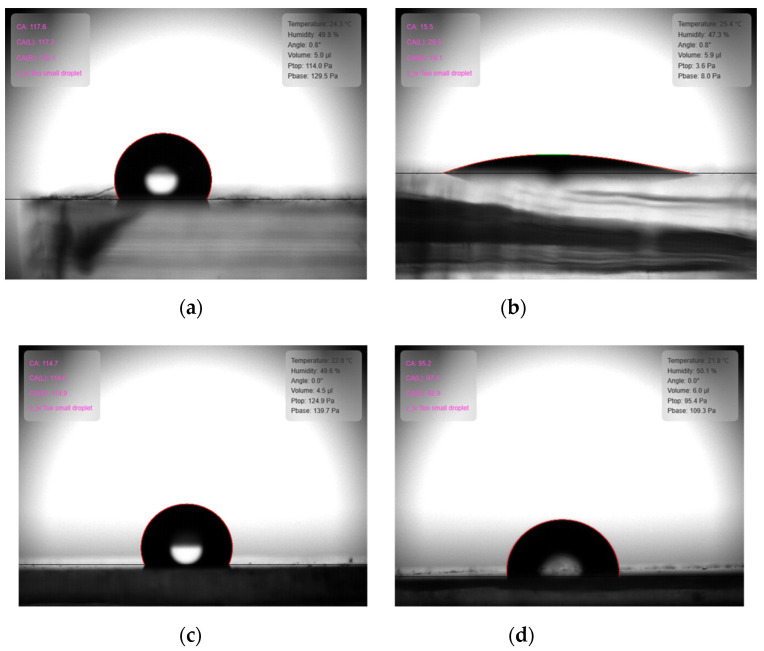
Comparison of contact angles: (**a**) pristine PDMS; (**b**) PDMS after O_2_ plasma treatment; (**c**) PANI growth on PDMS after O_2_ plasma treatment; and (**d**) PANI growth on PDMS after O_2_ plasma and APTES treatments.

The contact angle (CA) is a good indicator of the hydrophilicity or hydrophobicity of the surface. Figure 4 shows the contact angles of DI water between the PDMS and PANI surfaces. Since the pristine PDMS surface has –CH_3_ functional groups, it is predicted that the surface is hydrophobic; thus, it is confirmed from the CA of over 110° that the pristine PDMS is hydrophobic (Figure 4a). From the O_2_ plasma treatment of the PDMS surface, it is seen that the PDMS surface becomes hydrophilic with a CA of less than 20° (Figure 4b). However, after O_2_ plasma treatment and PANI polymerization, the surface returns to the hydrophobic state with a CA over 110°, similar to that of pristine PDMS (Figure 4c). Thus, it is seen that the surface of PANI is also hydrophobic like that of PDMS. The O_2_ plasma treatment process is hence required for good APTES deposition on the PDMS surface; when the CA is measured after completing APTES treatment and PANI synthesis, it is confirmed that the PANI surface becomes hydrophobic with a CA over 90° (Figure 4d).

### 3.4. Strain and I–V Characteristics

**Figure 5:**
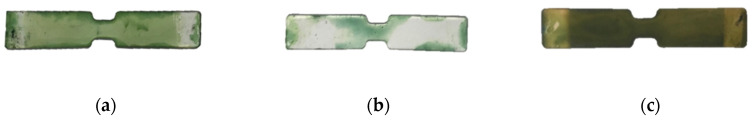
PANI growth on PDMS: (**a**) PANI on pristine PDMS; (**b**) PANI on PDMS after O_2_ plasma treatment; (**c**) PANI on PDMS after O_2_ plasma and APTES treatments.

PANI can be formed in three types as leucoemeraldine, emeraldine, and pernigraniline through the synthesis processes and represents the base and salt structures via redox reactions in each form. One of the characteristics of PANI is that it shows different colors depending on the synthesized form. Among these, the emeraldine salt structure having conductivity exhibits a green color. Figure 5 illustrates the synthesis of PANI on PDMS substrate, and all three illustrations above show a green color and conductivity; thus, it is expected that PANI would have an emeraldine salt structure. Figure 5a shows PANI synthesized on PDMS without any treatment, and Figure 5b shows PANI synthesis after O_2_ plasma treatment. Compared with synthesis on pristine PDMS, when a plasma treatment is performed, the coverage is significantly lowered by the PDMS surface property, which become hydrophilic owing to the –OH groups. However, as in Figure 5c, when PANI synthesis is performed after O_2_ plasma and APTES treatment with –NH_2_ functional groups, the coverage is confirmed to be better than that after plasma treatment of the pristine surface. The –NH_2_ functional group is hydrophilic similar to the –OH group, but both PANI and APTES have the same –NH_2_ functional groups, which offer advantages in the synthesis process.

**Figure 6:**
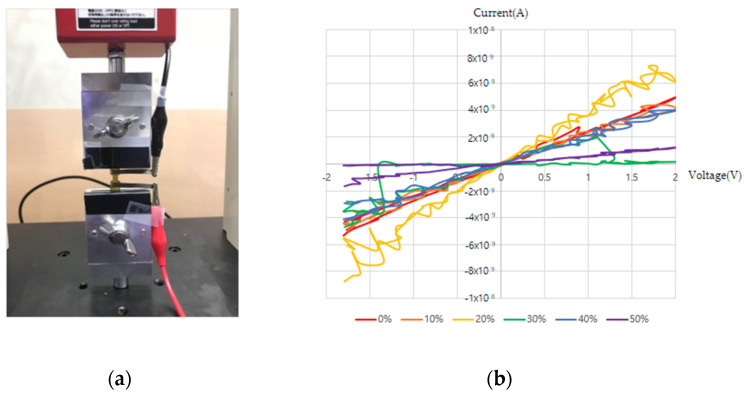

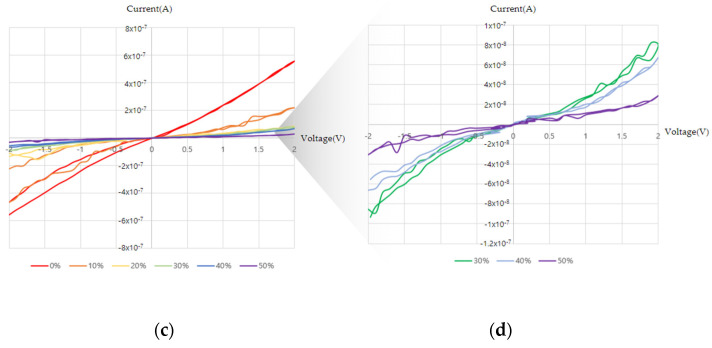
I–V characteristics: (**a**) connection to the tensile machine; (**b**) PANI growth on pristine PDMS; (**c**) PANI growth on PDMS after O_2_ plasma and APTES treatments; (**d**) magnified view of PANI growth characteristics on PDMS after O_2_ plasma and APTES treatments.

We conducted I–V measurements according to the strain for PANI on pristine PDMS and PANI on PDMS after O_2_ plasma and APTES treatments. PANI on PDMS after O_2_ plasma treatment is judged to have not been polymerized on the substrate because of significantly lower coverage than those of the other samples, thereby showing the characteristics of poor conductor; hence, I–V measurements were not shown here. Figure 6a shows the current measurements performed by attaching nickel tape and varying the voltage from −2 V to 2 V in steps of 0.1 V with dual sweeping. The strain was assessed for 0%, 10%, 20%, 30%, 40% and 50%, and Figure 6b–d shows the I–V values according to each strain. Figure 6b shows I–V trends according to strain of the PANI polymerization sample for pristine PDMS. When strain was 0%, it showed about 5 × 10^−9^ A at 2 V, and as seen from the graph, it was not possible to ensure stable current values based on the voltage and strain values. Figure 6c shows the I–V measurements for PANI on PDMS after O_2_ plasma and APTES treatments, which has a value of 6 × 10^−7^ A at 2 V with 0% strain, and it was possible to obtain stable measurements depending on the voltage. In addition, when the strain was gradually increased from 0% to 50%, the current decreased, and when the maximum tensile value was 50%, a current of about 3 × 10^−8^ A was obtained. It should be noted here that a stable current value was seen in accordance with the voltage value for voltages of 0 V to 2 V, and the current value tended to decrease in accordance with 0% to 50% strain.

**Figure 7:**
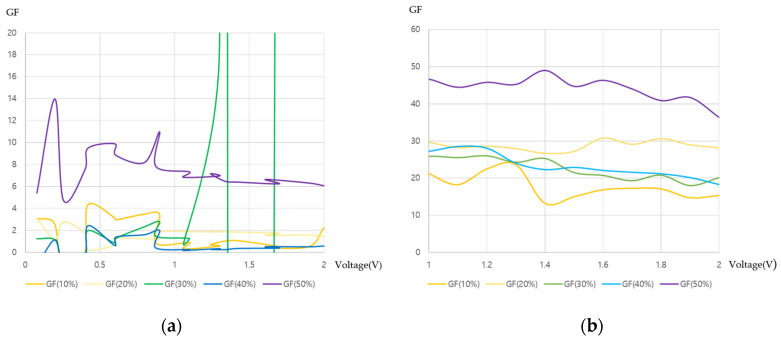

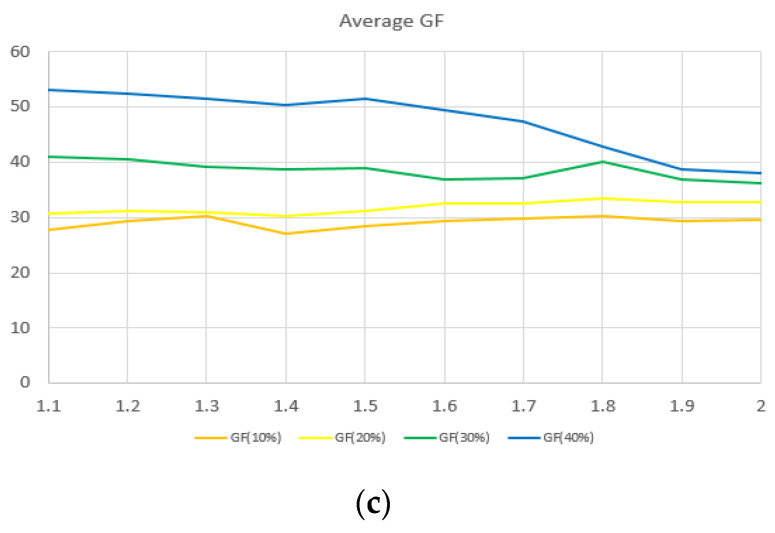
Gauge factor: (**a**) PANI growth on pristine PDMS; (**b**) PANI growth on PDMS after O_2_ plasma and APTES treatments; (**c**) average of three PANI samples on PDMS after O_2_ plasma and APTES treatments.

Figure 7 shows the GF values. In Figure 7a, the GF is in the range of 6–14 when the strain is 50%; this is not only a low value for a strain sensor but also shows poor stability of the sample measuring at different strains. However, from Figure 7b, a GF of 12–30 is confirmed when the strain is 10%, and a high GF of 38–49 is available when the strain is 50%. Relatively high and stable GF values can be secured. Additionally, we prepared three more samples after O_2_ plasma and APTES treatments. Although the GF value at 50% strain is not able to measure, average GF values with the strain from 10 to 40% are more stable according to the strain as shown in Figure 7c. Thus, the PANI synthesis on PDMS after APTES treatment of the strain sensor produces higher sensitivity and reliability than pristine PDMS.

## 4. Discussion

Various applications using flexible substrates are being commercialized and various materials are being developed. Among them, there are different process methods to utilize the good characteristics of PANI. However, the synthesis of PANI on PDMS is a well-known process, but it is not easy to obtain stable properties by directly synthesizing PANI on PDMS substrates with high uniformity. The process of bonding two different substances without any intermediate treatment is not easy when considering intermolecular interconnections. In order to obtain stable properties by combining heterogeneous substances, a surface treatment process must be accompanied. In this study, we propose a process to obtain higher uniformity and reliable operational characteristics than previously known PANI synthesis on PDMS through chemical surface treatment. In particular, the surface treatment method using a SAM is a very important part of synthesizing a conductive polymer on a flexible substrate. The result of PANI on PDMS synthesis without any surface treatment and the result of PANI on PDMS synthesis through surface treatment were compared. Finally, the PANI synthesis on the PDMS strain sensor after O_2_ plasma and APTES treatments produced a maximum GF of 49 at 50% strain, which shows that this configuration obtained higher coverage and better electrical characteristics than PANI synthesis on a pristine PDMS strain sensor. High GF at a high strain rate was secured, which is considered to be a good indicator in using PANI and PDMS in the future. We believe that strain sensors manufactured using surface treatments to synthesize conductive polymers can be used in various applications, such as wearables, electronic skin, and robotics.

## Figures and Tables

**Table 1:** Three different samples with PANI on PDMS.

	PANI	O_2_ Plasma	APTES
Sample 1	O	X	X
Sample 2	O	O	X
Sample 3	O	O	O
